# Supplementary material for: Biogeographic regionalization by spatial and environmental components: Numerical proposal
Source: PLoS One. 2021 Jun 15;16(6):e0253152. doi: 10.1371/journal.pone.0253152 (PMC8205180; doi:10.1371/journal.pone.0253152)
Supplement: S1 Table — (DOCX) [file pone.0253152.s001.docx]

**S1 Table. Variables used for the selection of environmental predictors in the seasonally dry tropical forest of the Balsas Depression, Mexico.**

| **Type** | **Variable** |
| --- | --- |
| *Climatic* | Bio02 (Mean diurnal range) |
|  | Bio03 (Isothermality) |
|  | Bio04 (Seasonality of Temperature) |
|  | Bio05 (Maximum temperature of the warmest month) |
|  | Bio12 (Annual rainfall) |
|  | Bio14 (Precipitation of Driest Month) |
|  | Bio15 (Seasonality of precipitation) |
|  | Bio17 (Precipitation of Driest Quarter) |
|  | Bio18 (Precipitation of the warmest quarter) |
|  | Bio19 (Precipitation of the coldest quarter) |
|  | evaanual (Annual real evapotranspiration) |
|  | Evasecos (Real evapotranspiration of dry months) |
| *Topographic* | Aspect (Orientation 0° to 90°) |
|  | Convergin (Convergence index) |
|  | Dah (Diurnal anisotropic heating) |
|  | Mexdem (Digital model of elevation) |
|  | Mexslope (Slope) |
|  | Runoff (Flow) |
|  | Twi (Topographic moisture index) |
|  | Vrm (Vector’s rugosity measure) |
| *Edaphic*  Cruz-Cárdenas et al. (2014) | Mexca (Calcium) |
|  | Mexco (Organic carbon) |
|  | Mexk (Potassium) |
|  | Mexna (Sodium) |
|  | Mexmg (Magnesium) |
|  | Mexmo (Organic material) |
|  | Mexph (Ph) |
|  | Mexras (Sodium absorption radius) |
| **MODIS* | Modisdic (Normalized vegetation index December) |
|  | Modismay (Normalized vegetation index May) |
|  | Modisabr (Normalized vegetation index April) |
|  | hum_modis2009 (Spectroradiometer wet months 2009) |

* Variables obtained with remote perception data (MODIS web): Moderate Resolution Imaging Spectroradiometer; December, February, March, and April 2009.
