# Supplementary material for: Biogeographic regionalization by spatial and environmental components: Numerical proposal
Source: PLoS One. 2021 Jun 15;16(6):e0253152. doi: 10.1371/journal.pone.0253152 (PMC8205180; doi:10.1371/journal.pone.0253152)
Supplement: S2 Table — (DOCX) [file pone.0253152.s002.docx]

**S2 Table. Characteristic species of phytogeographic groupings of Fig 3.**

| **Groups** | **Species** | **Biogeographic districts** |
| --- | --- | --- |
| 1 | *Aldama kingii, Melampodium tenellum, Packera bellidifolia, Sigesbeckia jorullensis.* | Lower Balsas |
| 2 | *-----* | Lower Balsas |
| 3 | *Acourtia bravohollisiana, A. cordata, A. cuernavacana, A. dugesii, A. glomeriflora, A. humboldtii, A. lepidopoda, A. lobulata, A. ovatifolia, A. reticulata, A. turbinata, Adenophyllum cancellatum, A. glandulosum, Ageratina bellidifolia, A. brevipes, A. cremasta, A. dolichobasis, A. espinosarum, A. glabrata, A. isolepis, A. josepaneroi, A. liebmannii, A. ligustrina, A. ramireziorum, A. rubricaulis, A. tomentella, Ageratum conyzoides, A. tehuacanum, Aldama buddleiiformis, A.a excelsa, A. hypochlora, A. morelensis, A. pachycephala, Alomia alata, Alloispermum michoacanum, Ambrosia confertiflora, Aphanostephus ramosissimus, Archibaccharis asperifolia, A. auriculata, A. schiedeana, A. serratifolia, Astranthium xylopodum, Axiniphyllum corymbosum, Baccharis conferta, B. sordescens, Bartlettina oresbia, Bidens alba ,B. aurea , B. bigelovii, B. gracillima, B. gypsophila, B. serrulata, Brickellia eupatorioides, B. filipes, B. huahuapana, B. jimenezii, B. oliganthes, B. paniculata, B. scoparia, B. secundiflora, B. tomentella, Calyptocarpus vialis, C. wendlandii, Carminatia alvarezii, C. tenuiflora, Centaurea rothrockii, Cirsium conspicuum, C. ehrenbergii, C. mexicanum, C. rhaphilepis, Conyza coronopifolia, C. sumatrensis, Coreopsis petrophiloides, C. rhyacophila, Cosmos caudatus, Cymophora pringlei, Chaptalia hintonii, C. nutans, C. salicifolia, Chromolaena glaberrima, Chrysactinia mexicana, Chrysanthellum involutum, Dahlia apiculata, D. cordifolia, D. merckii, D. parvibracteata, Dendroviguiera eriophora, D. guerrerana, D. insignis, D. oaxacana, Desmanthodium ovatum, Digitacalia cripta, D. jatrophoides, Dyssodia pinnata, Elephantopus mollis, Erechtites hieracifolius, Eremosis macvaughii, Eremosis tomentosa, Erigeron delphinifolius, E. tephropodus, Eryngiophyllum pinnatisectum, Flaveria angustifolia, F. trinervia, Fleischmannia holwayana, Florestina platyphylla, Flourensia glutinosa, Galinsoga triradiata, Gamochaeta americana, Gymnolaena oaxacana, Heliopsis buphthalmoides, Hieracium abscissum, Hymenostephium woronowii, Hymenothrix greenmanii, Hymenoxys integrifolia, Iostephane heterophylla, I. trilobata, Isocoma veneta, Laennecia filaginoides, Lasianthaea squarrosa, Leiboldia guerreroana, Liabellum hintoniorum, Megaliabum andrieuxii, Melampodium montanum, M. repens, Microspermum debile, M. michoacanum, Montanoa leucantha, M. mollissima, M. revealii, M. tomentosa, Otopappus imbricatus, O. verbesinoides, Packera toluccana, Parthenium bipinnatifidum, P. tomentosum, Pectis repens, P. saturejoides, Perymenium discolor, P. episcopale, P. mendezii, P. rogmacvaughii, Piqueria pilosa, Pittocaulon praecox, Porophyllum calcicola, P. coloratum, P. linaria, P. warnockii, Psacaliopsis paneroi, Psacalium amplifolium, P. guerreroanum, P. matudae, P. mollifolium, P. peltatum, Pseudognaphalium bourgovii, P. canescens, P. oxyphyllum, P. purpurascens, P. semilanatum, P. viscosum, Pseudogynoxys chenopodioides, Roldana angulifolia, R. barba-johannis, R. ehrenbergiana, R. eriophylla, R. hederifolia, R. lobata, R. platanifolia, R. suffulta, Salmea oligocephala, S. scandens, Sanvitalia fruticosa, Senecio cinerarioides, S. deformis, S. deppeanus, Simsia amplexicaulis, S. rhombifolia, Sinclairia glabra, S. moorei, Stevia clinopodioides, S. deltoidea, S. elatior, S. hypomalaca, S. incognita, S. jorullensis, S. lita, S. lucida, S. microchaeta, S. mitopoda, S. neurophylla, S. pilosa, S. porphyrea, S. seemannioides, S. serrata, S. suaveolens, S. triflora, S. viscida, Steviopsis amblyolepis, S. vigintiseta, Symphyotrichum potosinum, Tagetes coronopifolia, T. jaliscensis, T. persicifolius, T. tenuifolia, Tridax brachylepis, Trigonospermum annuum, Trixis calcicola, T. pringlei, Verbesina abscondita, V. auriculata, V. breedlovei, V. gigantea, V. gracilipes, V. hypomalaca, V. klattii, V. myriocephala, V. oaxacana, V. oncophora, V. serrata, V. virgata, Vernonia bealliae, Wedelia hintoniorum, W. purpurea, Xanthium strumarium, Zaluzania montagnifolia, Z. pringlei, Zandera andersoniae, Zinnia purpusii.* | Upper Balsas |
| 4 | *Adenophyllum aurantium, Ageratina oligocephala, Aldama perennans, Alloispermum longiradiatum, Bidens acrifolia, B. esmartinezii, Bidens hintonii, Cirsium subcoriaceum, Cosmos mattfeldii, Chrysanthellum filiforme, C. keilii, C. michoacanum, Decachaeta incompta, Heliopsis brachactis, Hymenostephium hintonii, Mikania micrantha, Pectis decemcarinata, P. diffusa, P. multiflosculosa, Perymenium hintonii, Roldana sessilifolia, Simsia tenuis, Stevia micradenia, Trixis hyposericea, Wamalchitamia strigosa, Wedelia keilii, Zinnia zinnioides.* | Lower Balsas |
| 5 | *Acourtia simulata, Ageratina geminata, A. macvaughii, Archibaccharis hirtella, Astranthium orthopodum, Bidens pringlei, Carphochaete grahamii, Dahlia sorensenii, Hieracium hintonii, Kyrsteniopsis nelsonii, Montanoa andersonii, Psacalium palmeri, P. silphiifolium, Pseudognaphalium stramineum, Roldana glinophylla, Stevia serrata, Tagetes foetidissima, Verbesina furfuracea, Zandera blakei.* | Upper Balsas |
| 6 | *Archibaccharis hieracioides, Cosmos nitidus, C. schaffneri, Roldana langlassei.* | Upper Balsas |
| 7 | *Flaveria kochiana, Vernonia karvinskiana.* | Upper Balsas |
| 8 | *Sinclairiopsis klattii* | Upper Balsas |
