# Supplementary material for: Biogeographic regionalization by spatial and environmental components: Numerical proposal
Source: PLoS One. 2021 Jun 15;16(6):e0253152. doi: 10.1371/journal.pone.0253152 (PMC8205180; doi:10.1371/journal.pone.0253152)
Supplement: Attachment — Submitted filename: PONE-D-21-01262.docx [file pone.0253152.s003.docx]

**Comments**

This study highlights the district level biogeographic regionalization of the Asteraceae species in the Balsas regions of Mexico. Study is very interesting and innovative highlighting different environment variables to be responsible for varied species richness and their distribution. This study has potential for identifying smaller biotic regions for endemic and other ecologically important species to conserve and can be replicated in similar regions. However, few points are of concern here and are summarized below to be addressed by the authors.

**Abstract:**

**P2-L42:** differ between them,…. Replace with ‘differ significantly’

**P2-L43:** than in the Upper Balsas**…** Shouldn’t it be ‘lower Balsas’ ? Please check

**Introduction:**

**P3-L59:** in terms of their endemic taxa….. I wonder if this regionalization meant to be specific for endemic taxa? Its always in broader terms referring to biota. Please recheck.

**P4-L109:** regionalization …….BD. insert ‘of’ before ‘the SDTF’.

**P5-L115:** The regionalization………..Replace ‘DB’ with ‘BD’.

**Materials and methods:**

**Spatial data**

**P5-L143:** [55]: 1) This is confusing. Please insert ‘as:’ before 1)

As per point no.1 How did you geo-reference the points with no co-ordinates? Please clear.

**P6-L154:** ‘Environmental variables’. Should you be consistent with the terms i.e. variables/predictors?

**P7-L182-184:** We used the dissimilarity…….Biodiverse program. This looks redundant with the previous paragraph (P6 L160, L165-166). Could you try to club these together and put extra information in the sentence?

**Ordination analysis**

**P7-L195:** along of reduced ….. Replace ‘of’ with ‘the’.

**P7-L201-202:** We extracted…… NMDS. Replace ‘ArcGis’ with ‘ArcGIS’.

**P7-L202:** Should you add any figure no. for refence?

**Selection of SDTF environmental predictors**

**P7-L204:** First we considered……… (S1 Table). This is confusing as I observed there are only 32 uncorrelated variables and not 58 in S1. Therefore mention the ‘S1 table’ reference after uncorrelated variables (P8-L214).

**Results**

**P8-L234-235:** The differentiated…….. species. Rephrase the sentence i.e. the differentiated groups shown in the dendrogram (Fig 3, a) represent exclusive species of the groups (S2 Table).

**P8-L234:** Replace ‘Fig 3’ with ‘(Fig 3, a)’.

**P8-L229-230; P9-L251-257:** Although……… Balsas; The biogeographic tracks…… composition. Be consistent with the use of words. i.e. in the MS mostly groups and districts are used alternatively which might be confusing for the readers with abrupt appearance in the paragraph.

**P9-L253-257:** Each identified……. Altitudes. Same as previous comment. The sentence becomes very confusing for the common readers because of the use of alternative terminology e.g. lower Balsas or track. Insert ‘in the upper Balsas’ after eastern track.

**SDTF environmental predictors**

**P10-L270:** The most parsimonious …….. Here 10 variables are mentioned however, in the table 1 only 9 variables are shown. Please confirm.

**Discussion**

**P11-L297:** Our results….. Rephrase the sentence i.e. Our results are in congruence with……

**P11-L297:** Replace ‘DB’with ‘BD’.

**P11-L309:** Remove ‘BD’.

**P11-L312:** These results…….. China. Is the study mentioned represent similar region i.e. SDTF. Also the China study highlights the richness correlation with annual precipitation however in the Balsas the dominance of herbs (58%) might be influenced by seasonal environmental variable as rightly captured in this study. You may use other reference for this.

**P12-L334-335:** It has been….. (Fig 4). Should you replace the “(Fig 4)” with (Fig 3)? Since there is no descriptive label to represent group 3.

**Figures**

**Figure 3 b:** Could you add labels for group ? i.e. Group 3: Upper Balsas District etc. for readers understanding.

**Table**

S1 table: Are there no representative species in group 2? How it was delineated as separate district based on the analysis?
